# Neurotoxicity associated with the medicinal mushroom product-Diamond Shruumz: A case report

**DOI:** 10.1016/j.toxrep.2024.101748

**Published:** 2024-09-23

**Authors:** Elizabeth F. Ebbens, Saralyn R. Williams, Rebecca E. Bruccoleri, S. Barron Frazier

**Affiliations:** a Pediatrics, Pediatric Emergency Medicine, Monroe Carell Jr. Children's Hospital at Vanderbilt, 2200 Children's Way, Nashville, TN 37212, USA; b Emergency Medicine, Vanderbilt University Medical Center, 2215 Garland Ave, Nashville, TN 37232, USA; c Medicine, Clinical Pharmacology, Vanderbilt University Medical Center, 1161 21st Ave, Nashville, TN 37232, USA

**Keywords:** Mushroom(s), Edible(s), Neurotoxin(s), Ingestion, Toxidrome, Myoclonic jerks, Mydriasis, Neurotoxicity

## Abstract

Medicinal mushrooms are widely available as health supplements, and the federal government does not currently require these products to be examined for quality and contents. This places consumers at risk for unintentional ingestion of other substances, including toxic mushroom species. We describe a case report of an ingestion of an edible medicinal mushroom product likely contaminated with muscimol, the primary toxin of *Amanita muscaria*. A 17-year-old female presented with altered mental status, mydriasis, salivation, and myoclonic jerks that were refractory to benzodiazepines. She was intubated for airway protection and had spontaneous improvement of all her symptoms with return to baseline within 8 hours of presentation. She disclosed ingestion of the chocolate bar brand “Diamond Shruumz” that has been recalled for muscimol contamination. She was discharged home the day after presentation without symptom recurrence. This case displays the toxidrome of muscimol ingestion consistent with prior reports in the literature from muscimol containing mushroom ingestion. To our knowledge, this is among the first reports of *Amanita muscaria* ingestion from a commercially available medicinal mushroom product.

## Highlights

• An adolescent female presented with lethargy, mydriasis, sialorrhea, and myoclonus.
• She had eaten a medicinal mushroom product contaminated with *Amanita muscaria*.
• *Amanita muscaria* contains the neurotoxin muscimol.
• Muscimol ingestion may result in a mixed cholinergic and antimuscarinic toxidrome.

## Graphical Abstract

**Figure:**
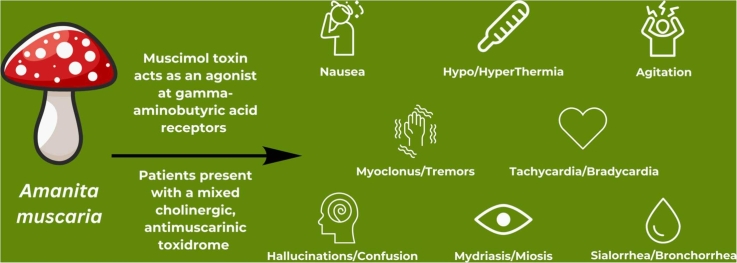

## 1 Introduction

Medicinal mushrooms are growing in popularity as supplements with purported health benefits and psychoactive properties. These include mushrooms such as Reishi, Chaga, Lion’s Mane, and Shiitake. Historically used in Eastern medicine as a therapeutic for a variety of diseases, emerging research on medicinal mushrooms has demonstrated a myriad of health benefits from cancer prevention to fighting infections [1], [2]. In the United States, regulated therapeutics containing these mushrooms are available only in the context of clinical trials. Other formulations are sold as dietary supplements or edibles, whose contents are not regulated by the Food and Drug Administration (FDA) for ingredients and dose [2]. Supplements purporting to contain a specific mushroom may place consumers at risk for unintended ingestions of other substances. There is a particular risk of contamination with toxic mushroom species and toxins, specifically those of the genus *Amanita*.

## 2 Case report

In late June 2024, a 17-year-old female arrived at the pediatric emergency department at Monroe Carell Jr. Children’s Hospital at Vanderbilt at 1 AM with altered mental status via ambulance. She was found by a family member at home with jerking movements of her upper and lower extremities. Upon arrival, her vital signs were within normal limits for age. She was placed on continuous cardiac monitoring and end tidal carbon dioxide monitoring. Physical examination revealed an ill appearing teenage female. Her neurologic examination revealed opening eyes to noxious stimuli, mumbling intermittently. Pupillary exam showed 5 mm pupils that were equal and minimally reactive. She had myoclonic jerks of all extremities. She would withdraw from painful stimuli and have sustained, intensified myoclonus. She had 4+ reflexes throughout and her ankles were held in dorsiflexion. Her jaw was clenched shut and she was noted to have profuse watery oral secretions. She had a normal cardiopulmonary exam. Her abdomen was soft and nontender. She had strong pulses throughout with capillary refill < 2 seconds. Twelve lead electrocardiogram obtained shortly after arrival showed sinus rhythm with normal intervals. Initial venous blood gas (VBG) measured normal pH, or electrolytes, lactate, carboxyhemoglobin andor methemoglobin levels.

The patient received one-liter intravenous crystalloid fluids and a four-milligram dose of ondansetron intravenously. A head computed tomography scan revealed no acute intracranial abnormalities. Laboratory analyses including complete metabolic panel, complete blood count, thyroid stimulating hormone, thyroxine, creatine kinase, salicylate levels, acetaminophen levels, alcohol levels and urine drug screen was obtained. Her evaluation revealed only an abnormal urine drug screen that was positive for tetrahydrocannabinol (THC). Two milligrams of midazolam were given intravenously with only a transient improvement in myoclonus. She required intubation given inability to protect her airway. She was admitted to the Intensive Care Unit (ICU) for ongoing care.

Four hours after her initial presentation, at approximately 5 AM, the patient became more alert and responded to commands. She was extubated approximately 3 hours later with resolution of her myoclonus. She disclosed to ICU providers that earlier in the evening, she consumed a Diamond Shruumz Cookies and Cream chocolate bar that she received as a free sample from a vape shop. She also reported weekly use of THC gummies but none in the 24 hours prior to presentation. She only recalled eating the mushroom product and then waking in the hospital hours later.

Toxicology discussed with the medical team that the Centers for Disease Control was currently investigating an outbreak of poisonings related to these specific therapeutic mushroom-containing products, which had been recalled for containing the muscimol toxin of *Amanita muscaria* in addition to other natural and synthetic psychoactive compounds. The patient was transferred to the hospital floor and discharged the following day without complication. Toxicology providers sent an expanded toxicology screen was negative for fentanyl metabolites, benzodiazepine metabolites (apart from known Midazolam) or other co-ingestions.

## 3 Discussion

This case represents one of over 150 reported cases in a documented outbreak of medicinal mushroom products contaminated with toxic mushroom compounds [3]. Prior cases of muscimol toxicity have usually been described in association with ingestion of the *Amanita muscaria* mushroom itself rather than an occult ingestion with an medicinal mushroom product. The association between the patient’s presentation and muscimol toxicity is supported by the ingestion of substance known to contain muscimol in addition to both cholinergic (bronchorrhea) and antimuscarinic (dilated pupils) symptoms with involuntary muscle contractions and rigidity. The temporal effect of this substance also fits well given the rapid onset and short duration of symptoms, less than 8 hours from the time of ingestion.

Of the multiple hallucinogenic mushroom species, *Amanita muscaria*, also known as “fly agaric” or the “Alice in Wonderland” mushroom, is known for its stereotypical red cap with white scales [4], [5]. *Amanita muscaria* primarily causes central nervous system toxicity, specifically a Type 2 C neurotoxicity according to the classification proposed by White et al. in 2019 [6]. Though muscarine, an acetylcholine agonist, gives the mushroom its name, it is only present in minuscule amounts in *Amanita muscaria* and has little effect on the toxidrome [5]. The more abundant toxigenic compounds include ibotenic acid, a neuroexcitatory molecule structurally similar to glutamic acid, and muscimol, a gamma-aminobutyric acid (GABA) agonist [5].

After absorption, these molecules enter the bloodstream and pass through the blood brain barrier. Once in the brain, ibotenic acid is converted to muscimol by glutamate decarboxylase, making muscimol the primary mediator of *Amanita* effects [3], [4]. Binding to GABA receptors, it causes fluctuating neurologic symptoms with an initial period of drowsiness followed by confusion, visual disturbances and hallucinations, agitation, ataxia, myoclonus, and tremors. Nausea, vomiting, and diarrhea may occur. Both cholinergic and antimuscarinic effects have also been observed including miosis/mydriasis, hypothermia/ hyperthermia, and tachycardia/bradycardia. Bronchorrhea is rare [5], [6], [7], [8]. Patients may have amnesia after ingestion [6]. Poisoning presents within minutes up to 3 hours from ingestion. Symptoms last up to 48 hours, but more often 24 hours or less [6], [7]. Rat models of muscimol toxicity show an LD50 dose of 42 mg per kg−1 (intravenous) and 129 mg per kg−1 (oral) [4], [7]. Muscimol doses as low as 6 mg, or a single *Amanita muscaria* cap have caused symptoms in humans [4], [8]. There is no antidote to ingestion, only supportive care for symptoms [8].

Review articles and case reports describing muscimol toxicity have reported sequalae of direct ingestion of *Amanita muscaria* or other similar *Amanita* species. A case series from 2012 to 2016 describes thirty-four cases of ingestion of *Amanita* species containing ibotenic acid with symptoms as described above [8]. Most poisonings are related to foraging errors or intentional ingestions for psychotropic effects. Patients often required benzodiazepines for supportive care and only three of the thirty-four required intubation [8]. A review of reports to the National Poison Data Center from 1999 to 2016 identified 751 cases of ingestion of ibotenic acid containing mushrooms with only one fatality [9]. Fatality is exceedingly rare as most cases require little to no intervention [9].

Our patient exhibited some of the typical neuroexcitatory effects of muscimol ingestion including confusion and myoclonus, as well as post-ingestion amnesia. Additionally, her case was notable for excess oral secretions and mydriasis with a mixed cholinergic and anti-muscarinic response. Her case exhibited severe features from many described previously in the literature, as she required intubation for airway protection. Many of her symptoms do correlate with muscimol ingestion with a confirmed source, however, this report is limited as there may also be other toxins in the specific product not yet identified. This may have contributed to the severity of her symptoms and the mix of both cholinergic and antimuscarinic symptoms, although certainly these have all been described in the setting of muscimol ingestion alone.

## 4 Conclusions

As more products containing medicinal or hallucinogenic mushrooms come to market, the public should be aware of the risks of taking these products, including the risk for co-ingestion of toxic substances. Our patient’s case demonstrates the neurotoxicity of a mushroom product likely contaminated with muscimol, the primary neurotoxic molecule in *Amanita muscaria*.

## CRediT authorship contribution statement

**Elizabeth F Ebbens:** Writing – original draft, Conceptualization. **Steven Barron Frazier:** Writing – review & editing, Supervision, Conceptualization. **Rebecca Bruccoleri:** Supervision. **Saralyn Williams:** Writing – review & editing.

## Declaration of Competing Interest

The authors declare that they have no known competing financial interests or personal relationships that could have appeared to influence the work reported in this paper.

## Data Availability

No data was used for the research described in the article.
